# Systematic Comparison of Three Commercially Available Combination Disc Tests and the Zinc-Supplemented Carbapenem Inactivation Method (zCIM) for Carbapenemase Detection in *Enterobacterales* Isolates

**DOI:** 10.1128/JCM.03140-20

**Published:** 2021-08-18

**Authors:** Janko Sattler, Anne Brunke, Axel Hamprecht

**Affiliations:** a University of Cologne, Faculty of Medicine, Cologne, Germany; b University Hospital Cologne, Institute for Medical Microbiology, Immunology and Hygiene, Cologne, Germany; c German Centre for Infection Research (DZIF), Partner Site Bonn‐Cologne, Cologne, Germany; d Institute for Medical Microbiology and Virology, University of Oldenburg, Oldenburg, Germany; Medical College of Wisconsin

**Keywords:** combination disc testing, carbapenem resistance, zCIM, multiresistant *Enterobacterales*, faropenem, KPC, NDM, OXA-48, carbapenemase inactivation method

## Abstract

Detection of carbapenemases in *Enterobacterales* is crucial for patient treatment and infection control. Among others, combination disc tests (CDTs) with different inhibitors (e.g., EDTA) and variations of the carbapenem inactivation method (CIM) are recommended by EUCAST or the CLSI and are used by many laboratories as they are relatively inexpensive. In this study, we compare three commercially available CDTs, faropenem disc testing (FAR), and the zinc-supplemented CIM (zCIM) test for the detection of carbapenemase-producing *Enterobacterales* (CPE). The Rosco KPC/MBL and OXA-48 Confirm kit (ROS-CDT), the Liofilchem KPC&MBL&OXA-48 disc kit (LIO-CDT), Mastdiscs Combi Carba plus (MAST-CDT), FAR, and zCIM were challenged with 106 molecularly characterized CPE and 47 non-CPE isolates. The sensitivities/specificities were 86% (confidence interval [CI], 78 to 92%)/98% (CI, 89 to 100%) for MAST-CDT and ROS-CDT, 96% (CI, 91 to 99%)/87% (CI, 74 to 95%) for LIO-CDT, and 99% (CI, 95 to 100%)/81% (CI, 67 to 91%) for FAR compared to 98% (CI, 93 to 100%)/100% (CI, 92 to 100%) for zCIM. The CDTs showed great performance differences depending on the carbapenemase class, with MAST-CDT and LIO-CDT best detecting class B, ROS-CDT best detecting class A, and LIO-CDT best detecting class D carbapenemases. The overall performance of commercially available CDTs was good but varied greatly for different carbapenemases and between manufacturers, compared with FAR and zCIM, which performed well for all carbapenemase types. For reliable carbapenemase detection, CDTs should preferably not be used as the sole test but can be part of a diagnostic strategy when combined with other assays (e.g., CIM-based, immunochromatographic, or molecular tests).

## INTRODUCTION

Antimicrobial resistance has been recognized as one of the top 10 threats to public health by the World Health Organization (1). Among multiresistant bacteria, carbapenemase-producing *Enterobacterales* (CPE) show the most alarming development in recent years (2). Due to the rapid spread within and between species via horizontal gene transfer, the global spread of carbapenemases has led to an increasing prevalence of CPE worldwide (3), limiting therapeutic options for antimicrobial treatment (4).

While there is a broad consensus that the early detection of CPE is crucial for patient treatment and infection control (5), the increasing numbers of tests for carbapenemase detection with varying performances, costs, turnaround times, and laboratory requirements are a challenge for diagnostic laboratories (6). In their latest guidelines, the European Committee on Antimicrobial Susceptibility Testing (EUCAST) recommends phenotypic testing for carbapenemases on *Enterobacterales* isolates that show elevated MICs or reduced disc diffusion zone diameters compared to the screening cutoff values for ertapenem and/or meropenem (7). Among the recommended phenotypic tests are combination disc tests (CDTs), the carbapenem inactivation method (CIM), colorimetric tests, immunochromatographic lateral flow assays, and tests based on mass spectrometry (7). The Clinical and Laboratory Standards Institute (CLSI) recommends testing for carbapenemase production in special situations, e.g., for epidemiology or infection control purposes. In these cases, the recommended tests are CarbaNP, a colorimetric test, the modified carbapenem inactivation method (mCIM), and/or molecular assays (8).

Combination disc tests are among the first tests that have been used for the detection of carbapenemases in diagnostic laboratories, as they are inexpensive and relatively easy to perform. They utilize the ability of certain chemical compounds to specifically inhibit carbapenemases from different Ambler classes. Boronic acid inhibits class A carbapenemases (serine carbapenemases), and EDTA and dipicolinic acid inhibit class B carbapenemases (metallo-β-lactamases [MBLs]). As there is no specific inhibitor for class D carbapenemases (oxacillinases), the presence of high-level temocillin resistance in the absence of inhibition by boronic acid and dipicolinic acid or EDTA is the most common way to detect this carbapenemase class (9). In-house preparation of CDTs is generally feasible, with results comparable to those of commercially available tests (10). However, due to quality control requirements, this is cumbersome to introduce and maintain in the diagnostic laboratory. As an easier alternative, several companies have developed ready-to-use CDTs, which are easy to implement and perform.

Another disc diffusion-based method to screen for CPE is faropenem disc-based testing (FAR) (11). This test can detect CPE by the absence of a faropenem inhibition zone (class A or B carbapenemases) or exhibition of a double inhibition zone (class D carbapenemases). Among the tests with a turnaround time similar to that of CDTs is the zinc-supplemented carbapenem inactivation method (zCIM), which is a variation of the CLSI-recommended mCIM. Compared to other CIM variations, the detection of MBLs is improved with zCIM (12). The test principle is based on the hydrolysis of meropenem in a meropenem disc, which is incubated in a zinc-supplemented bacterial suspension. Following hydrolysis by a carbapenemase, no growth inhibition is observed around the meropenem disc when placed on a lawn of bacteria of a wild-type Escherichia coli strain.

This study aims to compare the latest CDTs of three manufacturers together with FAR and zCIM on a large-scale set of molecularly characterized CPE isolates.

## MATERIALS AND METHODS

The following CDTs were included in this study: Mastdiscs Combi Carba plus (MAST-CDT; Mast Diagnostica, Reinfeld, Germany), the KPC/MBL and OXA-48 Confirm kit (ROS-CDT; Rosco, Taastrup, Denmark), and the KPC&MBL&OXA-48 disc kit (LIO-CDT; Liofilchem, Roseto degli Abruzzi, Italy). Additionally, the CAT-ID test (FAR; Mast Diagnostica), which consists of a 10-μg faropenem disc, and the zCIM test were evaluated.

All tests were challenged with 106 CPE and 47 non-CPE isolates (controls). The isolates comprised those with a broad spectrum of carbapenemases, including the most prevalent types KPC, NDM, VIM, IMP, and OXA-48-like along with the rarer carbapenemase types IMI, GES, and OXA-58, and three isolates with two carbapenemases. CPE isolates included Klebsiella pneumoniae, E. coli, Enterobacter cloacae complex, Citrobacter freundii complex, Serratia marcescens, Proteus mirabilis, Klebsiella oxytoca, and Raoultella ornithinolytica (see Table S1 in the supplemental material). The presence of carbapenemases was confirmed using immunochromatographic assays, PCR, and Sanger sequencing as previously described (12, 13). Phenotypic characterization of isolates for extended-spectrum β-lactamase (ESBL) and AmpC production was carried out as previously described (13, 14).

Additionally, whole-genome sequencing (WGS) data, acquired with short-read sequencing technology (Illumina), were used for genomic analyses of selected isolates. Data analysis was carried out as previously described (15, 16). Abricate software (17) was employed to match sequences with the ResFinder database (18) to search for resistance genome determinants.

Combined disc tests were performed according to the manufacturers’ recommendations. Briefly, a bacterial suspension with a turbidity equivalent to a 0.5 McFarland standard was prepared from fresh bacterial isolates and inoculated onto Mueller-Hinton (MH) agar (MHA) (Oxoid, Basingstoke, UK). After 5 to 10 min, the combination discs were placed on the inoculated plates. After incubation at 37°C for 18 h, inhibition zones were recorded. Microcolonies within a clearly delineated inhibition zone (Fig. S1) were ignored for the measurements in the analyses for all CDTs as recommended in the MAST-CDT manual.

A significant increase of inhibition zone size between the combination of a specific carbapenemase inhibitor plus carbapenem and the carbapenem alone indicated a positive result for the corresponding carbapenemase class. As boronic acid inhibits both class A carbapenemases and class C β-lactamases, the carbapenem-cloxacillin combination disc, which inhibits only class C β-lactamases, had to be interpreted in order to differentiate between these two classes. If inhibitor-carbapenem combinations did not show a significant increase of the inhibition zone, the diameter of the temocillin inhibition zone was evaluated for the presence of class D carbapenemases. Cutoff values for the different CDTs are listed in Table S2.

If none of the listed criteria applied, the isolate was interpreted as being carbapenemase negative. Isolates carrying more than one carbapenemase were included in the overall sensitivity calculation of the assay but not in the subclass analysis, as results cannot be clearly interpreted according to the schemes provided by the manufacturers.

Additionally, FAR, which is not included in the original MAST-CDT kit, was evaluated in this study. If no inhibition zone or a double inhibition zone was observed around the faropenem disc, the test was considered positive. Otherwise, the test was considered negative.

In cases where a false-positive or false-negative result or an incorrect carbapenemase class was identified, isolates were retested on three different MH agars, from (i) Oxoid, (ii) Axonlab (Stuttgart, Germany), and (iii) Becton, Dickinson (Heidelberg, Germany), to check for agar-specific differences. If results were discrepant between the initial test and the repetition using Oxoid MHA, a third test was performed to decide the final result.

For quality control purposes, each batch of CDTs was tested with E. coli ATCC 25922 (negative control) and a positive control for each Ambler class (C. freundii KPC-3, E. coli NDM-3, and E. coli OXA-244). Positive controls were obtained from the National Reference Centre for multidrug-resistant Gram-negative bacteria.

The zCIM test was performed as described previously (12) but with 1.5 mM ZnSO_4_ instead of 0.3 mM ZnSO_4_ because an increase in the sensitivity of 16% was demonstrated in a pilot study on 19 CPE when the higher ZnSO_4_ concentration was used. Carbapenemase activity in isolates producing VIM-58 (*n* = 2) and VIM-4 (*n* = 1) was detected only with the higher zinc concentration. For test preparation, a full 10-μl inoculation loop of bacteria grown on MHA (Oxoid) was suspended in 400 μl of tryptic soy broth supplemented with 1.5 mM ZnSO_4_. A meropenem 10-μg disc (I2A, Montpellier, France) was submerged and incubated at 37°C for 2 h. Subsequently, the disc was transferred onto MHA (Oxoid) inoculated with a suspension of ATCC 25922 E. coli cells equivalent to a 0.5 McFarland standard. After 18 h of incubation at 37°C, the inhibition zone was measured. An inhibition zone of ≤20 mm was interpreted as positive, and an inhibition zone of >20 mm was interpreted as negative.

For each test, screening sensitivity, Ambler class-specific sensitivity, specificity, 95% confidence intervals (CIs) (exact Clopper-Pearson confidence intervals), and the Youden index were calculated. Thereby, screening sensitivity refers to carbapenemase detection in CPE regardless of whether the correct Ambler class of the carbapenemase was detected.

Prism 8.1 (GraphPad, San Diego, CA, USA) and Excel (Microsoft, Redmond, WA, USA) were used for statistical analyses. Continuous variables were assessed by a Mann-Whitney U test. A *P* value of <0.05 was considered significant.

## RESULTS

### Performances of combination disc tests and temocillin and faropenem disc tests.

Carbapenemases were detected in 91/106 CPE (86% [CI, 78 to 92%]) for MAST-CDT and ROS-CDT and 102/106 CPE (96% [CI, 91 to 99%]) for LIO-CDT (Table 1). Of 47 controls, 46 were correctly classified as negative by MAST-CDT and ROS-CDT, and 41 were correctly classified as negative by LIO-CDT (Table 1). However, some isolates gave a positive result for a carbapenemase but not for the correct Ambler class. Excluding the CPE with more than one carbapenemase, correct classifications according to Ambler class were recorded for 88/103 CPE (85% [CI, 77 to 92%]) for MAST-CDT, 86/103 (84% [CI, 75 to 90%]) for ROS-CDT, and 99/103 (96% [CI, 90 to 99%]) for LIO-CDT (Table 2).

**TABLE 1:** Screening sensitivities, specificities, and Youden indices for the different tests^*a*^

Parameter	Value for test				
	MAST-CDT	ROS-CDT	LIO-CDT	FAR	zCIM
No. of true-positive isolates/total no. of isolates	91/106	91/106	102/106	105/106	104/106
Sensitivity (%) (CI)	86 (78–92)	86 (78–92)	96 (91–99)	99 (95–100)	98 (93–100)
No. of true-negative isolates/total no. of isolates	46/47	46/47	41/47	38/47	47/47
Specificity (%) (CI)	98 (89–100)	98 (89–100)	87 (74–95)	81 (67–91)	100 (92–100)
Youden index	0.84	0.84	0.83	0.80	0.98

a CI, confidence interval; MAST-CDT, Mastdiscs Combi Carba plus; ROS-CDT, Rosco KPC/MBL and OXA-48 Confirm kit; LIO-CDT, Liofilchem KPC&MBL&OXA-48 disc kit; FAR, Mast CAT-ID; zCIM, zinc-supplemented carbapenem inactivation method.

**TABLE 2:** Ratios of correctly identified carbapenemases and Ambler class-specific sensitivities of combination disc testing^*a*^

Ambler class	MAST-CDT		ROS-CDT		LIO-CDT	
	No. of correct identifications/total no. of isolates	Sensitivity (%) (CI)	No. of correct identifications/total no. of isolates	Sensitivity (%) (CI)	No. of correct identifications/total no. of isolates	Sensitivity (%) (CI)
All	88/103	85 (77–92)	86/103	83 (75–90)	99/103	96 (90–99)


A	19/29	66 (46–82)	29/29	100 (88–100)	28/29	97 (82–100)
GES	0/1	0 (0–98)	1/1	100 (3–100)	1/1	100 (3–100)
IMI	7/9	78 (40–97)	9/9	100 (66–100)	8/9	89 (52–100)
KPC	12/19	63 (38–84)	19/19	100 (82–100)	19/19	100 (82–100)

B	45/48	94 (83–99)	32/48	67 (52–80)	45/48	94 (83–99)
IMP	4/4	100 (40–100)	3/4	75 (19–99)	3/4	75 (19–99)
NDM	26/27	96 (81–100)	21/27	78 (56–91)	27/27	100 (87–100)
VIM	15/17	88 (64–99)	8/17	47 (23–72)	15/17	88 (64–99)

D	24/26	92 (75–99)	25/26	96 (80–100)	26/26	100 (87–100)
OXA-48	6/6	100 (54–100)	5/6	83 (36–100)	6/6	100 (54–100)
OXA-48-like	17/18	94 (73–100)	18/18	100 (81–100)	18/18	100 (81–100)
OXA-58	1/2	50 (1–99)	2/2	100 (16–100)	2/2	100 (16–100)

a CI, confidence interval; MAST-CDT, Mastdiscs Combi Carba plus; ROS-CDT, Rosco KPC/MBL and OXA-48 Confirm kit; LIO-CDT, Liofilchem KPC&MBL&OXA-48 disc kit. OXA-48-like includes OXA-162, -181, -232, -244, -245, and −370.

Carbapenemase subgroup analysis showed that each CDT exhibits particular strengths and weaknesses in the detection of carbapenemases from different Ambler classes. While MAST-CDT and LIO-CDT correctly detected 94% (CI, 83 to 99%) of the class B carbapenemases, ROS-CDT correctly detected 100% (CI, 88 to 100%) of the class A carbapenemases, and LIO-CDT correctly detected 100% (CI, 87 to 100%) of the class D carbapenemases (Table 2).

For three isolates carrying a single carbapenemase, synergism with both boronic and dipicolinic acids was observed with ROS-CDT (strains K5, K19, and K48 in Table 3). These were an IMI-14-producing E. cloacae isolate, a KPC-2-producing Citrobacter braakii isolate, and an NDM-1-producing S. marcescens isolate. MAST-CDT showed double synergism (KPC inhibitor and MBL inhibitor) for the same NDM-1-producing S. marcescens isolate. However, no double synergism was observed for all three isolates by LIO-CDT, which uses EDTA as an MBL inhibitor. Additionally, WGS analysis did not identify any additional carbapenemase genes for the C. braakii and S. marcescens isolates. The NDM-1-producing S. marcescens isolate carried CMY-6, which is also inhibited by boronic acid compounds, which subsequently resulted in a KPC phenotype with ROS-CDT and MAST-CDT. No WGS data were available for the E. cloacae isolate.

**TABLE 3:** Individual results for each isolate tested^*a*^

Strain	Species	Carbapenemase(s)	MIC (μg/ml)			zCIM result	zCIM diam (mm)	MAST-CDT result	ROS-CDT result	LIO-CDT result	FAR result	Other β-lactamase gene(s)
			EPM	IPM	MEM							
K1	Citrobacter freundii	GES-25	>32	>32	>32	Pos	6	Neg	KPC	KPC	NIZ	*bla*_CMY-63_, *bla*_OXA-2_, *bla*_TEM-3_, *bla*_OXA-1_
K2	Enterobacter cloacae	IMI-1	2	>32	1	Pos	6	KPC	KPC	KPC	NIZ	None
K3	Enterobacter cloacae	IMI-2	>32	>32	>32	Pos	6	KPC	KPC	KPC	NIZ	None
K4	Enterobacter cloacae	IMI-12	>32	>32	>32	Pos	6	KPC	KPC	Neg	NIZ	None
K5	Enterobacter cloacae	IMI-14	8	>32	8	Pos	6	Neg	KPC	KPC	NIZ	NA
K6	Enterobacter cloacae	IMI-16	>32	>32	>32	Pos	15	Neg	KPC	KPC	NIZ	*bla*_ACT-10_
K7	Enterobacter cloacae	IMI-2	>32	>32	>32	Pos	6	KPC	KPC	KPC	NIZ	*bla*_ACT-9_
K8	Enterobacter cloacae	IMI-3	8	16	4	Neg	21	KPC	KPC	KPC	NIZ	*bla*_ACT-12_
K9	Enterobacter cloacae	IMI-4	16	>32	16	Pos	6	KPC	KPC	KPC	NIZ	*bla*_MIR-2_
K10	Enterobacter cloacae	IMI-9	>32	>32	16	Pos	6	KPC	KPC	KPC	NIZ	None
K11	Enterobacter cloacae	KPC-2	>32	>32	>32	Pos	6	KPC	KPC	KPC	NIZ	*bla*_TEM-1B_, *bla*_OXA-1_, *bla*_ACT-5_
K12	Enterobacter cloacae	KPC-2	16	>32	8	Pos	6	KPC	KPC	KPC	NIZ	*bla*_TEM-1B_, *bla*_OXA-1_, *bla*_ACT-5_
K13	Klebsiella pneumoniae	KPC-2	>32	>32	>32	Pos	6	OXA-48	KPC	KPC	NIZ	*bla*_OXA-9_, *bla*_TEM-122_, *bla*_SHV-12_
K14	Klebsiella pneumoniae	KPC-2	16	32	>32	Pos	6	KPC	KPC	KPC	NIZ	*bla*_CTX-M-15_, *bla*_OXA-9_, *bla*_SHV-145_
K15	Klebsiella pneumoniae	KPC-2	>32	>32	>32	Pos	6	Neg	KPC	KPC	NIZ	*bla*_SHV-12_, *bla*_TEM-1A_, *bla*_OXA-9_
K16	Klebsiella pneumoniae	KPC-2	>32	>32	>32	Pos	6	Neg	KPC	KPC	NIZ	*bla*_SHV-182_, *bla*_OXA-9_, *bla*_TEM-122_
K17	Klebsiella pneumoniae	KPC-2	32	32	32	Pos	6	Neg	KPC	KPC	NIZ	*bla*_OXA-9_, *bla*_TEM-122_, *bla*_SHV-187_
K18	Klebsiella pneumoniae	KPC-2	>32	>32	>32	Pos	6	Neg	KPC	KPC	NIZ	*bla*_OXA-9_, *bla*_TEM-122_, *bla*_SHV-187_
K19	Citrobacter braakii	KPC-2	>32	32	32	Pos	6	KPC	KPC	KPC	NIZ	*bla*_TEM-1B_, *bla*_OXA-1_
K20	Klebsiella pneumoniae	KPC-2	>32	>32	>32	Pos	6	Neg	KPC	KPC	NIZ	*bla*_SHV-182_, *bla*_OXA-9_, *bla*_TEM-122_
K21	Klebsiella pneumoniae	KPC-2	>32	16	>32	Pos	6	KPC	KPC	KPC	NIZ	*bla*_SHV-12_, *bla*_TEM-122_, *bla*_OXA-9_
K22	Klebsiella pneumoniae	KPC-2	>32	>32	>32	Pos	6	KPC	KPC	KPC	NIZ	*bla*_SHV-182_, *bla*_OXA-9_, *bla*_TEM-122_
K23	Klebsiella pneumoniae	KPC-3	>32	>32	>32	Pos	6	KPC	KPC	KPC	NIZ	*bla*_SHV-106_, *bla*_CTX-M-15_, *bla*_OXA-1_
K24	Klebsiella pneumoniae	KPC-3	4	8	4	Pos	6	KPC	KPC	KPC	NIZ	NA
K25	Klebsiella pneumoniae	KPC-3	>32	32	>32	Pos	6	KPC	KPC	KPC	NIZ	*bla*_SHV-106_, *bla*_CTX-M-15_, *bla*_OXA-1_
K26	Klebsiella pneumoniae	KPC-3	>32	>32	>32	Pos	6	KPC	KPC	KPC	NIZ	*bla*_SHV-182_, *bla*_OXA-9_, *bla*_TEM-122_
K27	Citrobacter freundii	KPC-3	>32	>32	>32	Pos	6	Neg	KPC	KPC	NIZ	*bla*_SHV-182_, *bla*_TEM-1B_, *bla*_OXA-10_, *bla*_CTX-M-15_, *bla*_OXA-1_, *bla*_CMY-48_
K28	Citrobacter freundii	KPC-3	2	4	2	Pos	6	KPC	KPC	KPC	NIZ	*bla*_CMY-65_, *bla*_TEM-1A_, *bla*_OXA-9_
K29	Citrobacter freundii	KPC-3	2	>32	2	Pos	6	KPC	KPC	KPC	NIZ	*bla*_CMY-65_, *bla*_TEM-1A_, *bla*_OXA-9_
K30	Klebsiella pneumoniae	IMP-1	>32	8	16	Pos	6	MBL	MBL	MBL	NIZ	*bla*_SHV-110_, *bla*_CTX-M-15_, *bla*_TEM-1B_, *bla*_OXA-1_
K31	Klebsiella pneumoniae	IMP-22	>32	>32	>32	Pos	6	MBL	Neg	Neg	NIZ	*bla*_OXA-2_, *bla*_TEM-1A_, *bla*_SHV-106_, *bla*_OXA-9_
K32	Klebsiella pneumoniae	IMP-4	4	1	4	Pos	6	MBL	MBL	MBL	NIZ	*bla*_DHA-1_, *bla*_TEM-1B_, *bla*_CTX-M-14_, *bla*_SHV-11_
K33	Citrobacter freundii	IMP-8	>32	>32	16	Pos	6	MBL	MBL	MBL	NIZ	*bla*_OXA-10_, *bla*_OXA-2_, *bla*_OXA-1_, *bla*_CMY-98_
K34	Klebsiella pneumoniae	NDM-1	>32	>32	32	Pos	6	MBL	MBL	MBL	NIZ	*bla*_SHV-182_, *bla*_CTX-M-15_, *bla*_OXA-1_
K35	Raoultella ornithinolytica	NDM-1	16	8	8	Pos	6	MBL	MBL	MBL	NIZ	*bla*_OXA-1_, *bla*_CTX-M-15_
K36	Klebsiella pneumoniae	NDM-1	>32	>32	>32	Pos	6	MBL	MBL	MBL	NIZ	*bla*_CMY-4_, *bla*_TEM-1B_, *bla*_SHV-182_, *bla*_CTX-M-15_
K37	Klebsiella pneumoniae	NDM-1	16	16	32	Pos	6	MBL	MBL	MBL	NIZ	*bla*_SHV-11_, *bla*_CTX-M-15_, *bla*_TEM-1B_, *bla*_OXA-1_
K38	Klebsiella pneumoniae	NDM-1	>32	4	8	Pos	6	MBL	MBL	MBL	NIZ	*bla*_SHV-182_, *bla*_CTX-M-15_, *bla*_OXA-1_
K39	Klebsiella pneumoniae	NDM-1	>32	>32	>32	Pos	6	MBL	MBL	MBL	NIZ	*bla*_SHV-182_
K40	Enterobacter cloacae	NDM-1	>32	32	32	Pos	6	MBL	MBL	MBL	NIZ	*bla*_OXA-10_
K41	Escherichia coli	NDM-1	4	2	4	Pos	6	MBL	Neg	MBL	DIZ	*bla*_CTX-M-15_, *bla*_TEM-1B_, *bla*_OXA-1_
K42	Enterobacter cloacae	NDM-1	>32	32	16	Pos	6	MBL	MBL	MBL	NIZ	*bla*_ACT-16_, *bla*_OXA-1_
K43	Enterobacter cloacae	NDM-1	32	>32	>32	Pos	6	MBL	MBL	MBL	NIZ	*bla*_OXA-1_, *bla*_ACT-16_
K44	Klebsiella pneumoniae	NDM-1	>32	>32	>32	Pos	6	MBL	MBL	MBL	NIZ	*bla*_SHV-11_, *bla*_CTX-M-15_, *bla*_TEM-1A_
K45	Escherichia coli	NDM-1	>32	>32	>32	Pos	6	MBL	MBL	MBL	NIZ	NA
K46	Escherichia coli	NDM-1	32	8	32	Pos	6	MBL	MBL	MBL	NIZ	*bla*_CMY-6_, *bla*_TEM-1A_, *bla*_OXA-2_
K47	Escherichia coli	NDM-1	>32	>32	>32	Pos	6	MBL	MBL	MBL	NIZ	*bla*_CMY-6_, *bla*_TEM-1A_, *bla*_OXA-2_
K48	Serratia marcescens	NDM-1	>32	>32	>32	Pos	6	Neg	KPC	MBL	NIZ	*bla*_CMY-6_, *bla*_TEM-1B_, *bla*_SHV-12_
K49	Klebsiella pneumoniae	NDM-1	8	8	8	Pos	6	MBL	MBL	MBL	NIZ	*bla*_TEM-1A_, *bla*_SHV-11_, *bla*_OXA-9_, *bla*_CTX-M-15_, *bla*_OXA-1_
K50	Klebsiella pneumoniae	NDM-1	>32	>32	>32	Pos	6	MBL	MBL	MBL	NIZ	*bla*_CTX-M-15_, *bla*_OXA-9_, *bla*_OXA-1_, *bla*_TEM-1B_, *bla*_SHV-106_
K51	Escherichia coli	NDM-3	4	4	2	Pos	6	MBL	MBL	MBL	NIZ	*bla*_SHV-12_, *bla*_TEM-1B_
K52	Escherichia coli	NDM-4	32	16	32	Pos	6	MBL	Neg	MBL	NIZ	*bla*_CTX-M-24_, *bla*_CMY-148_, *bla*_TEM-1B_
K53	Escherichia coli	NDM-5	>32	>32	>32	Pos	6	MBL	Neg	MBL	NIZ	*bla*_TEM-1B_, *bla*_CTX-M-15_, *bla*_OXA-1_
K54	Escherichia coli	NDM-5	>32	>32	>32	Pos	6	MBL	MBL	MBL	NIZ	*bla*_CMY-2_, *bla*_CTX-M-15_, *bla*_OXA-1_, *bla*_TEM-1B_
K55	Citrobacter freundii	NDM-5	32	32	4	Pos	6	MBL	MBL	MBL	NIZ	*bla*_TEM-1B_, *bla*_CMY-70_, *bla*_CTX-M-3_
K56	Escherichia coli	NDM-7	>32	>32	>32	Pos	6	MBL	Neg	MBL	NIZ	*bla*_CTX-M-15_, *bla*_CMY-6_, *bla*_OXA-1_
K57	Enterobacter cloacae	NDM-7	>32	>32	>32	Pos	6	MBL	MBL	MBL	NIZ	*bla*_CTX-M-15_, *bla*_ACT-16_, *bla*_OXA-1_, *bla*_TEM-1B_
K58	Klebsiella pneumoniae	NDM-8	>32	32	32	Pos	6	MBL	Neg	MBL	NIZ	*bla*_SHV-145_, *bla*_CTX-M-15_, *bla*_TEM-1B_, *bla*_OXA-9_
K59	Klebsiella pneumoniae	NDM-9	8	8	4	Pos	6	MBL	MBL	MBL	NIZ	*bla*_CTX-M-15_, *bla*_SHV-11_, *bla*_TEM-1A_, *bla*_OXA-9_
K60	Klebsiella pneumoniae	NDM-9	8	4	4	Pos	6	MBL	MBL	MBL	NIZ	*bla*_CTX-M-15_, *bla*_OXA-9_, *bla*_SHV-11_
K61	Klebsiella pneumoniae	VIM-1	1	4	4	Pos	6	MBL	MBL	MBL	NIZ	NA
K62	Escherichia coli	VIM-1	0.25	4	0.5	Pos	6	MBL	Neg	MBL	NIZ	*bla*_TEM-1B_
K63	Escherichia coli	VIM-1	0.5	>32	16	Pos	6	MBL	Neg	MBL	NIZ	*bla*_TEM-106_
K64	Escherichia coli	VIM-1	0.064	4	2	Pos	6	MBL	Neg	MBL	NIZ	*bla*_TEM-1B_
K65	Citrobacter freundii	VIM-1	0.5	8	0.5	Pos	6	MBL	MBL	MBL	NIZ	*bla*_CMY-48_, *bla*_TEM-1B_
K66	Citrobacter freundii	VIM-4	1	0.5	0.25	Pos	6	MBL	Neg	MBL	NIZ	*bla*_CTX-M-9_, *bla*_OXA-10_, *bla*_CMY-50_
K67	Klebsiella pneumoniae	VIM-2	0.125	0.5	0.25	Pos	6	Neg	Neg	Neg	NIZ	None
K68	Enterobacter cloacae	VIM-26	>32	>32	>32	Neg	21	Neg	MBL	MBL	NIZ	*bla*_ACT-3_, *bla*_ACC-1_, *bla*_TEM-1B_, *bla*_OXA-10_
K69	Citrobacter freundii	VIM-31	0.5	1	0.5	Pos	6	MBL	MBL	MBL	NIZ	*bla*_CMY-49_, *bla*_TEM-1B_
K70	Enterobacter cloacae	VIM-26	>32	>32	>32	Pos	6	MBL	MBL	MBL	NIZ	*bla*_ACT-3_, *bla*_ACC-1_
K71	Klebsiella oxytoca	VIM-4	1	16	2	Pos	6	MBL	OXA-48	MBL	NIZ	*bla*_OXY-4-1_, *bla*_OXA-1_, *bla*_CTX-M-15_
K72	Enterobacter cloacae	VIM-4	0.125	4	0.5	Pos	6	MBL	MBL	MBL	NIZ	NA
K73	Enterobacter cloacae	VIM-4	8	1	1	Pos	6	MBL	Neg	MBL	NIZ	*bla*_ACT-16_, *bla*_CTX-M-9_
K74	Klebsiella pneumoniae	VIM-46	0.5	4	0.5	Pos	6	MBL	MBL	MBL	NIZ	*bla*_SHV-106_, *bla*_CTX-M-15_, *bla*_TEM-1B_
K75	Serratia marcescens	VIM-54	4	>32	>32	Pos	16	MBL	MBL	MBL	NIZ	*bla*_TEM-1B_, *bla*_CTX-M-3_
K76	Citrobacter freundii	VIM-58	1	2	0.25	Pos	6	MBL	Neg	MBL	NIZ	*bla*_CTX-M-3_, *bla*_CMY-152_, *bla*_TEM-1B_
K77	Enterobacter cloacae	VIM-58	0.5	1	0.125	Pos	6	MBL	Neg	Neg	NIZ	*bla*_LAP-2_, *bla*_CTX-M-9_, *bla*_ACT-15_
K78	Escherichia coli	OXA-162	2	1	0.5	Pos	6	OXA-48	OXA-48	OXA-48	DIZ	None
K79	Klebsiella pneumoniae	OXA-162	2	1	0.5	Pos	6	OXA-48	OXA-48	OXA-48	DIZ	*bla*_SHV-187_
K80	Klebsiella pneumoniae	OXA-162	32	8	32	Pos	6	OXA-48	OXA-48	OXA-48	DIZ	*bla*_SHV-106_, *bla*_CTX-M-15_, *bla*_OXA-1_
K81	Citrobacter freundii	OXA-162	4	2	1	Pos	6	OXA-48	OXA-48	OXA-48	DIZ	*bla*_OXA-1_, *bla*_CMY-152_, *bla*_TEM-1B_
K82	Escherichia coli	OXA-181	8	1	1	Pos	6	OXA-48	OXA-48	OXA-48	DIZ	*bla*_CTX-M-15_, *bla*_OXA-1_, *bla*_CMY-42_
K83	Escherichia coli	OXA-181	2	0.5	0.25	Pos	6	OXA-48	OXA-48	OXA-48	DIZ	*bla*_TEM-1B_, *bla*_CMY-42_, *bla*_CTX-M-24_
K84	Escherichia coli	OXA-181	2	0.5	0.5	Pos	6	OXA-48	OXA-48	OXA-48	DIZ	*bla*_CTX-M-15_, *bla*_OXA-1_
K85	Escherichia coli	OXA-181	2	0.25	0.25	Pos	8	OXA-48	OXA-48	OXA-48	DIZ	*bla*_CTX-M-15_, *bla*_OXA-1_, *bla*_TEM-1B_, *bla*_CMY-2_
K86	Klebsiella pneumoniae	OXA-162	32	8	32	Pos	6	OXA-48	OXA-48	OXA-48	DIZ	*bla*_TEM-1B_, *bla*_SHV-145_
K87	Klebsiella pneumoniae	OXA-232	>32	>32	>32	Pos	6	OXA-48	OXA-48	OXA-48	DIZ	*bla*_OXA-1_, *bla*_TEM-1A_, *bla*_CTX-M-15_, *bla*_SHV-106_
K88	Escherichia coli	OXA-181	4	0.5	0.25	Pos	6	OXA-48	OXA-48	OXA-48	NIZ	*bla*_OXA-1_, *bla*_CTX-M-15_, *bla*_TEM-35_
K89	Escherichia coli	OXA-232	2	0.5	0.25	Pos	6	OXA-48	OXA-48	OXA-48	DIZ	*bla*_CTX-M-15_, *bla*_TEM-1B_
K90	Escherichia coli	OXA-244	2	0.5	0.25	Pos	6	OXA-48	OXA-48	OXA-48	NIZ	*bla*_CTX-M-14b_, *bla*_TEM-1B_
K91	Klebsiella pneumoniae	OXA-244	32	32	32	Pos	6	OXA-48	OXA-48	OXA-48	DIZ	*bla*_CTX-M-15_, *bla*_OXA-1_, *bla*_SHV-110_
K92	Klebsiella pneumoniae	OXA-245	1	2	2	Pos	6	OXA-48	OXA-48	OXA-48	DIZ	*bla*_TEM-1B_, *bla*_SHV-182_, *bla*_CTX-M15_, *bla*_OXA-1_
K93	Klebsiella pneumoniae	OXA-245	4	32	32	Pos	6	OXA-48	OXA-48	OXA-48	DIZ	*bla*_SHV-182_
K94	Klebsiella pneumoniae	OXA-370	16	4	4	Pos	6	MBL	OXA-48	OXA-48	DIZ	*bla*_CTX-M-15_, *bla*_SHV-182_
K95	Escherichia coli	OXA-48	4	2	4	Pos	6	OXA-48	OXA-48	OXA-48	DIZ	*bla*_TEM-1B_, *bla*_CTX-M-24_
K96	Klebsiella pneumoniae	OXA-48	32	>32	>32	Pos	6	OXA-48	OXA-48	OXA-48	DIZ	*bla*_SHV-145_
K97	Escherichia coli	OXA-48	4	4	2	Pos	6	OXA-48	OXA-48	OXA-48	DIZ	None
K98	Escherichia coli	OXA-48	>32	8	>32	Pos	6	OXA-48	OXA-48	OXA-48	DIZ	*bla*_CTX-M-15_, *bla*_CMY-42_
K99	Klebsiella pneumoniae	OXA-48	>32	32	32	Pos	6	OXA-48	OXA-48	OXA-48	DIZ	*bla*_OXA-1_, *bla*_CTX-M-15_, *bla*_SHV-106_
K100	Escherichia coli	OXA-48	>32	1	2	Pos	6	OXA-48	Neg	OXA-48	DIZ	*bla*_CTX-M-122_, *bla*_TEM-1B_
K101	Proteus mirabilis	OXA-58	1	>32	16	Pos	6	OXA-48	OXA-48	OXA-48	DIZ	NA
K102	Proteus mirabilis	OXA-58	2	>32	4	Pos	6	OXA-48	OXA-48	OXA-48	Neg	None
K103	Klebsiella pneumoniae	KPC-2 + VIM-1	>32	>32	>32	Pos	6	Neg	OXA-48	OXA-48	DIZ	*bla*_SHV-11_
K104	Klebsiella pneumoniae	KPC-2 + VIM-1	>32	>32	>32	Pos	6	Neg	OXA-48	OXA-48	NIZ	*bla*_SHV-11_
K105	Klebsiella pneumoniae	OXA-232	>32	>32	>32	Pos	6	Neg	OXA-48	OXA-48	NIZ	*bla*_OXA-1_, *bla*_SHV-106_
K106	Escherichia coli	NDM-5 + OXA-181	8	>32	>32	Pos	6	MBL	MBL	MBL	NIZ	*bla*_CTX-M-15_, *bla*_TEM-1B_, *bla*_OXA-9_
K107	Enterobacter cloacae	Neg	1	0.5	0.25	Neg	23	Neg	Neg	Neg	Neg	*bla*_MIR-6_
K108	Klebsiella aerogenes	Neg	32	16	4	Neg	22	Neg	Neg	Neg	NIZ	None [AmpC^+^]
K109	Klebsiella aerogenes	Neg	32	4	1	Neg	24	Neg	KPC	Neg	NIZ	None [AmpC^+^]
K110	Proteus mirabilis	Neg	32	32	8	Neg	24	Neg	Neg	Neg	DIZ	*bla*_CMY-2_
K111	Enterobacter cloacae	Neg	1	2	0.25	Neg	25	Neg	Neg	Neg	Neg	NA
K112	Escherichia coli	Neg	0.008	0.25	0.016	Neg	24	Neg	Neg	Neg	Neg	[*bla*_CTX-M-1_]
K113	Klebsiella aerogenes	Neg	32	32	32	Neg	24	OXA-48	Neg	Neg	NIZ	None [AmpC^+^]
K114	Klebsiella pneumoniae	Neg	32	0.5	2	Neg	22	Neg	Neg	Neg	Neg	*bla*_SHV-106_, *bla*_TEM-1B_, *bla*_CTX-M-15_, *bla*_OXA-1_
K115	Enterobacter cloacae	Neg	8	1	1	Neg	24	Neg	Neg	Neg	DIZ	*bla*_ACT-14_
K116	Klebsiella aerogenes	Neg	2	1	0.25	Neg	25	Neg	Neg	OXA-48	Neg	None [AmpC^+^]
K117	Klebsiella pneumoniae	Neg	32	0.5	1	Neg	21	Neg	Neg	Neg	Neg	*bla*_SHV-1_
K118	Enterobacter cloacae	Neg	4	4	1	Neg	24	Neg	Neg	OXA-48	NIZ	*bla*_ACT-15_, *bla*_CTX-M-9_
K119	Klebsiella pneumoniae	Neg	8	8	1	Neg	24	Neg	Neg	Neg	DIZ	*bla*_SHV-145_, *bla*_CMY-2_
K120	Enterobacter cloacae	Neg	16	4	1	Neg	23	Neg	Neg	OXA-48	Neg	*bla*_TEM-1A_, *bla*_ACT-7_
K121	Citrobacter freundii	Neg	4	2	4	Neg	22	Neg	Neg	Neg	Neg	[AmpC^+^]
K122	Escherichia coli	Neg	16	0.5	2	Neg	24	Neg	Neg	Neg	Neg	*bla*_CTX-M-15_, *bla*_TEM-1B_
K123	Escherichia coli	Neg	32	2	4	Neg	25	Neg	Neg	OXA-48	Neg	[*bla*_TEM-1_]
K124	Escherichia coli	Neg	32	2	4	Neg	23	Neg	Neg	OXA-48	Neg	*bla*_TEM-1B_, *bla*_OXA-1_
K125	Enterobacter cloacae	Neg	32	8	4	Neg	22	Neg	Neg	Neg	Neg	*bla*_ACT-15_
K126	Escherichia coli	Neg	0.032	0.25	0.032	Neg	24	Neg	Neg	Neg	Neg	*bla*_TEM-1B_, *bla*_CTX-M-15_, *bla*_OXA-1_
K127	Klebsiella pneumoniae	Neg	32	0.5	2	Neg	24	Neg	Neg	Neg	Neg	*bla*_SHV-106_, *bla*_CTX-M-15_, *bla*_TEM-1B_
K128	Klebsiella aerogenes	Neg	32	16	4	Neg	24	Neg	Neg	Neg	Neg	None [AmpC^+^]
K129	Escherichia coli	Neg	>32	16	4	Neg	21	Neg	Neg	Neg	NIZ	*bla*_CMY-42_
K130	Escherichia coli	Neg	0.016	0.25	0.016	Neg	21	Neg	Neg	Neg	Neg	None
K131	Klebsiella aerogenes	Neg	>32	>32	8	Neg	24	Neg	Neg	Neg	NIZ	[AmpC^+^, ESBL^+^].
K132	Escherichia coli	Neg	0.016	0.125	0.016	Neg	23	Neg	Neg	Neg	Neg	[*bla*_CTX-M-15_]
K133	Enterobacter cloacae	Neg	0.032	0.25	0.032	Neg	24	Neg	Neg	Neg	Neg	[*bla*_CTX-M-1_]
K134	Klebsiella pneumoniae	Neg	0.032	0.25	0.032	Neg	26	Neg	Neg	Neg	Neg	NA
K135	Escherichia coli	Neg	0.032	0.5	0.064	Neg	23	Neg	Neg	Neg	Neg	[*bla*_CTX-M-3_]
K136	Klebsiella pneumoniae	Neg	4	0.25	4	Neg	22	Neg	Neg	Neg	Neg	*bla*_TEM-1B_, *bla*_OXA-9_
K137	Klebsiella pneumoniae	Neg	0.125	0.25	0.064	Neg	22	Neg	Neg	Neg	Neg	[*bla*_CTX-M-15_]
K138	Escherichia coli	Neg	0.064	0.25	0.032	Neg	23	Neg	Neg	Neg	Neg	[*bla*_CTX-M-15_]
K139	Enterobacter cloacae	Neg	0.064	0.5	0.064	Neg	24	Neg	Neg	Neg	Neg	[*bla*_SHV-ESBL(238S+240K)_, *bla*_TEM-1_]
K140	Escherichia coli	Neg	0.25	0.25	0.032	Neg	23	Neg	Neg	Neg	Neg	[*bla*_CTX-M-15_]
K141	Escherichia coli	Neg	0.064	0.25	0.032	Neg	25	Neg	Neg	Neg	Neg	[*bla*_CTX-M-15_]
K142	Escherichia coli	Neg	0.016	0.25	0.032	Neg	23	Neg	Neg	Neg	Neg	[*bla*_CTX-M-1_]
K143	Escherichia coli	Neg	0.016	0.25	0.032	Neg	25	Neg	Neg	Neg	Neg	[*bla*_CTX-M-1_]
K144	Escherichia coli	Neg	0.016	0.25	0.032	Neg	24	Neg	Neg	Neg	Neg	[*bla*_CTX-M-3_]
K145	Escherichia coli	Neg	0.016	0.25	0.032	Neg	23	Neg	Neg	Neg	Neg	[*bla*_CTX-M-15_]
K146	Klebsiella pneumoniae	Neg	0.5	0.5	0.25	Neg	24	Neg	Neg	OXA-48	Neg	[*bla*_CTX-M-15_]
K147	Escherichia coli	Neg	0.032	0.25	0.032	Neg	23	Neg	Neg	Neg	Neg	[*bla*_CTX-M-27_]
K148	Escherichia coli	Neg	0.032	0.125	0.032	Neg	23	Neg	Neg	Neg	Neg	[*bla*_CTX-M-15_]
K149	Escherichia coli	Neg	0.032	0.125	0.016	Neg	25	Neg	Neg	Neg	Neg	[*bla*_CTX-M-15_]
K150	Klebsiella pneumoniae	Neg	0.064	0.5	0.032	Neg	23	Neg	Neg	Neg	Neg	[*bla*_CTX-M-27_]
K151	Klebsiella pneumoniae	Neg	0.064	0.25	0.064	Neg	25	Neg	Neg	Neg	Neg	[*bla*_CTX-M-15_]
K152	Klebsiella pneumoniae	Neg	1	0.5	0.25	Neg	26	Neg	Neg	Neg	Neg	[*bla*_CTX-M-15_]
K153	Enterobacter cloacae	Neg	16	2	0.5	Neg	22	Neg	Neg	Neg	Neg	*bla*_ACT-9_

a MICs were determined by the Etest for ertapenem (EPM), imipenem (IPM), and meropenem (MEM). Results in brackets indicate results of phenotypic AmpC tests and PCR/Sanger sequencing of β-lactamase genes. MAST-CDT, Mastdiscs Combi Carba plus; ROS-CDT, Rosco KPC/MBL and OXA-48 Confirm kit; LIO-CDT, Liofilchem KPC&MBL&OXA-48 disc kit; FAR, Mast CAT-ID; zCIM, zinc-supplemented carbapenemase inactivation method; Pos, positive; Neg, negative; NIZ, no inhibition zone; DIZ, double inhibition zone; NA, no WGS sequence available.

The sensitivities of temocillin for the identification of class D carbapenemases were 96% (CI, 80 to 100%) for MAST-CDT and 100% (CI, 87 to 100%) for ROS-CDT and LIO-CDT. However, a temocillin inhibition zone below the cutoff was also recorded for class A or B carbapenemases in 13% (MAST-CDT), 30% (ROS-CDT), and 70% (LIO-CDT) of isolates. Hence, temocillin should be interpreted only if no synergy for class A or B carbapenemases is recorded, in order to avoid false-positive results for class D carbapenemases, as recommended by all manufacturers.

Using FAR, 105/106 (96% [CI, 95 to 100%]) of the CPE were correctly classified as carbapenemase positive. The specificity was only 81% (CI, 67 to 91%) (Table 1). One P. mirabilis isolate producing OXA-58 was misclassified as carbapenemase negative (K102 in Table 3). Differentiation of carbapenemase classes is not possible with this assay.

### Performance of the combination disc test in isolates producing two carbapenemases (*n* = 3).

One E. coli isolate producing NDM-5 and OXA-181 was correctly classified as positive for MBL and OXA-48-like by all CDTs, showing an increased inhibition zone for EDTA and dipicolinic acid, respectively, and no inhibition zone for temocillin. The two isolates carrying KPC-2 and VIM-1 showed no significantly increased inhibition zone for any of the carbapenem-inhibitor combinations. Depending on the temocillin inhibition zone result, this resulted in either false-negative results (MAST-CDT) or incorrect classification as class D carbapenemases (ROS-CDT and LIO-CDT) (K103 and K104 in Table 3).

All isolates that initially gave a false-negative or false-positive result on Oxoid MHA were assessed additionally on MHAs from Axonlab and BD. With a different MH agar, a correct result could be achieved in 11% of isolates with initially false-negative and in 12% with initially false-positive test results (see Table S3 in the supplemental material).

### Performance of zCIM.

**FIG 1:**
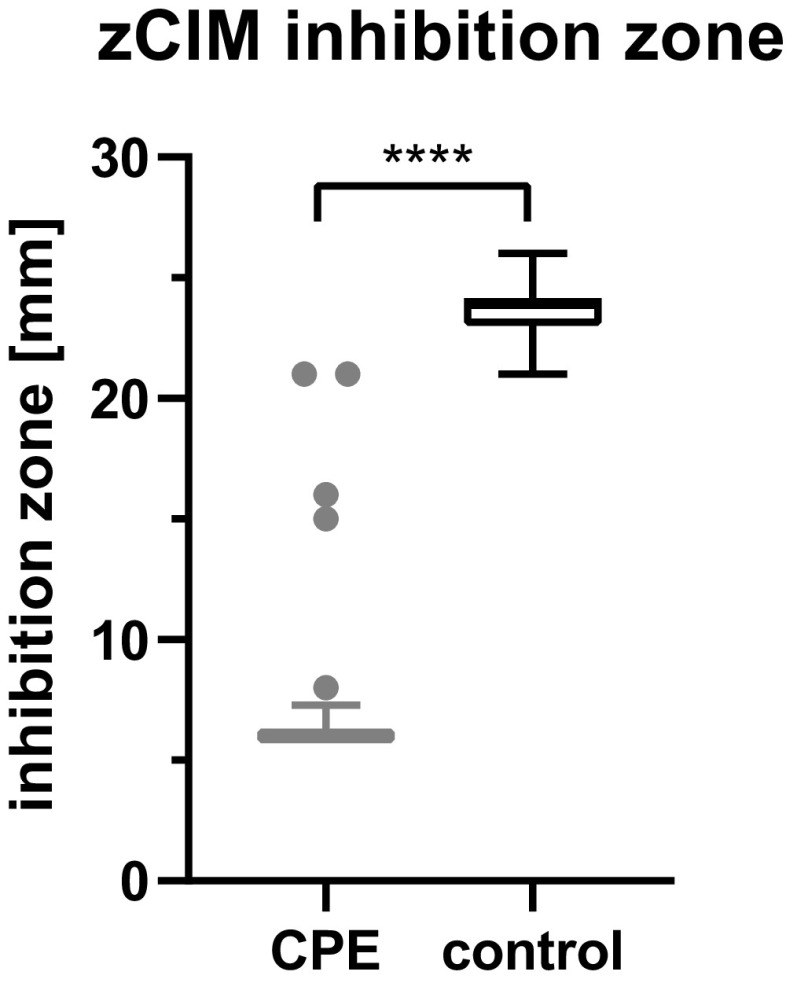
Box plot of the inhibition zone diameters of the zinc-supplemented carbapenem inactivation method (zCIM). Boxes represent quartiles, whiskers represent the 5th/95th percentiles, and dots represent outliers. Most carbapenemase-producing *Enterobacterales* (CPE) showed no inhibition zone (equal to a 6-mm diameter of the meropenem disc). Exceptions were an OXA-181-producing Escherichia coli isolate (8 mm), an IMI-16-producing Enterobacter cloacae isolate (15 mm), a VIM-54-producing Serratia marcescens isolate (16 mm), and the two CPE isolates that gave false-negative results. ****, *P* < 0.0001.

With zCIM, 104/106 (98% [CI, 93 to 100%]) CPE and 47/47 (100% [CI, 92 to 100%]) non-CPE isolates were correctly identified (Table 1). Two carbapenemase-producing E. cloacae isolates, one with IMI-3 carbapenemase and one with VIM-26, showed inhibition zones of 21 mm and were therefore slightly above the threshold for positive results (K8 and K68 in Table 3). The inhibition zone diameter was 23.5 mm for non-CPE isolates, compared to 6.5 mm for CPE isolates (*P* < 0.0001) (Fig. 1). Exceptionally, P. mirabilis isolates showed a swarming phenomenon from the bacterial colony remnants on the meropenem disc, which must be ignored for the measurement of the inhibition zone.

## DISCUSSION

This study systematically compared three different commercially available combination disc tests, faropenem disc testing, and zCIM for carbapenemase detection using a large selection of molecularly characterized CPE. All tests can be performed with standard equipment/media and are relatively inexpensive compared to immunochromatographic or molecular assays. They are therefore frequently used in diagnostic laboratories for carbapenemase detection.

Some CDTs have been evaluated previously; however, only a single or two different CDTs have usually been compared in previous studies. The ROS-CDT in its current form has been evaluated in several studies, with good sensitivity values of 90 to 100%, depending on the carbapenemase type, and a specificity of 92 to 93% (19, 20). Some studies also reported problems in the detection of CPE, particularly those with class D carbapenemases (21). In the present study, the CPE detection rates were slightly lower than those reported previously. Class D CPE detection rates were similar to those of the other classes. Rosco states in the manual that the identification of MBLs (particularly VIM-1) is difficult in isolates with meropenem inhibition zones of >25 mm. This was also observed in the present study, where six out of seven CPE with a meropenem inhibition zone of >25 mm were misclassified; these isolates produced VIM-1 (*n* = 2), VIM-58 (*n* = 2), VIM-2 (*n* = 1), and NDM-1 (*n* = 1). This largely contributed to the weak sensitivity of this CDT for class B carbapenemases.

The recent version of MAST-CDT has been evaluated on only a small collection of isolates with a limited range of carbapenemases (22, 23). While Ohsaki et al. reported a sensitivity of 100% for all carbapenemase types, Hu et al. described difficulties in detecting class A carbapenemases, with a sensitivity of 82%. In our study, these shortcomings could be demonstrated as well, with a sensitivity for class A carbapenemases of only 66%.

For the LIO-CDT, only one small-scale evaluation has been performed so far (24). While the reported sensitivity was 100% for class A and D carbapenemases, VIM carbapenemases could not be detected in that study. Indeed, in our study, the sensitivity for VIM carbapenemases was only 88%. Nevertheless, LIO-CDT showed by far the highest overall sensitivity (96%), but also the lowest specificity (87%), of all CDTs evaluated in this study.

The overall performances of the different CDTs varied greatly between isolates of different Ambler classes. If a laboratory decides to include a CDT in its screening process, we recommend basing the choice of a CDT on the local prevalence of carbapenemase subtypes. Class A carbapenemases are the most prevalent carbapenemases worldwide and in particular in the United States and southern parts of Europe (2). In these areas, the ROS-CDT might be preferred over the others as it detected 100% of the class A carbapenemases. In most parts of Asia, where class B carbapenemases are more prevalent (25), the MAST-CDT or LIO-CDT might be the better alternative. As the prevalence of class D carbapenemases is increasing worldwide (26), but particularly in Europe (27), the good performance of the LIO-CDT to detect OXA-48-like CPE might make it the most useful CDT there.

Most false-positive results were observed with the temocillin disc test (*n* = 7), using the LIO-CDT (*n* = 6) or MAST-CDT (*n* = 1). A low specificity of temocillin has been previously reported (28), especially in isolates with highly expressed AmpC (29). Indeed, 4/7 isolates were phenotypically positive for AmpC, and all belonged to species that typically hyperproduce AmpC (i.e., Klebsiella aerogenes and E. cloacae) (30). Additionally, in the two E. cloacae isolates, plasmidic AmpC *bla*_ACT-7/15_ was detected by WGS. The three other isolates that gave a false-positive result for OXA-48 were all phenotypically negative for AmpC expression. While one K. pneumoniae isolate was genotypically positive for CTX-M-15, TEM-1 was detected in the two E. coli isolates. Additionally, one false-positive result was recorded for KPC by ROS-CDT in a K. aerogenes isolate that was phenotypically AmpC positive.

The faropenem disc test showed a very good sensitivity of 99% in this study, which is in line with previous publications (11, 31). However, for even higher detection of class D CPE and higher specificity, the combination of FAR with temocillin should be used, as previously proposed (32). The combination of FAR and any of the CDTs evaluated in this study (all of which include temocillin) resulted in a sensitivity of 100%. Despite the lower specificity, this combination might be a useful screening tool that, in cases of positivity, should be confirmed/specified with other assays (e.g., immunochromatographic assays or PCR).

When isolates with false-positive/negative results were retested using a different MHA, minor changes in sensitivity and specificity in combination with certain CDTs were recorded (see Table S3 in the supplemental material). This phenomenon cannot be clearly attributed to a specific compound of the agar (e.g., zinc), as improved performance could be observed among carbapenemases of different classes. However, a thorough evaluation of this effect on all tested isolates for a series of MHA brands is beyond the scope of this study.

**FIG 2:**
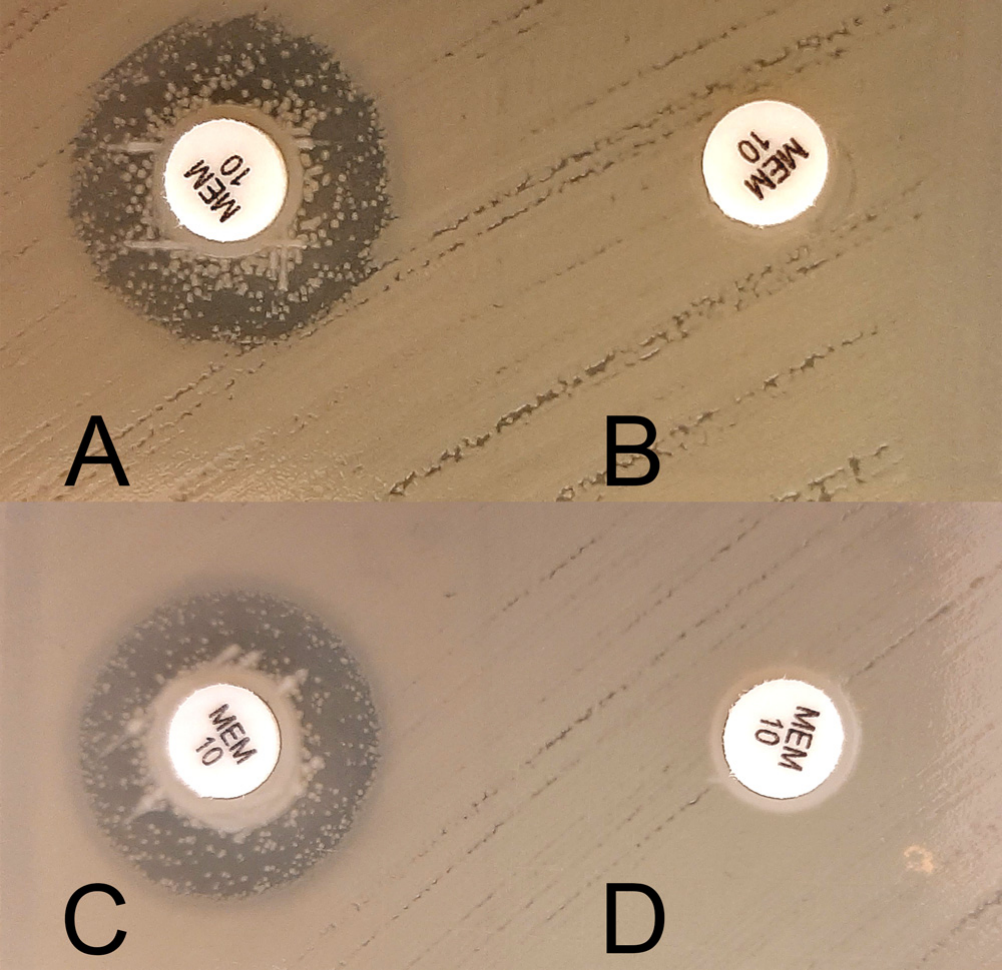
Illustrative example of the difference the between modified carbapenem inactivation method (mCIM) (A and C) and the zinc-supplemented carbapenem inactivation method (zCIM) (B and D) for metallo-β-lactamases. (A and B) Enterobacter cloacae isolate carrying VIM-26; (C and D) NDM-1-positive Serratia marcescens isolate. In these examples, both tests shown are interpreted as positive for the presence of a carbapenemase.

High sensitivity and specificity values were achieved with zCIM, as reported previously (12). Lower detection rates of the conventional mCIM for MBL can be overcome by supplementation with ZnSO_4_ in the tryptic soy broth medium (12) (Fig. 2). The greatest disadvantage of zCIM, as for the CDT evaluated here, is the long turnaround time of 18 to 20 h. Compared to mCIM, incubation in broth is 2 h shorter, and reading of some isolates with MBL is easier (Fig. 2). Recently, shorter incubation times of as little as 10 h have been reported for CIM, without a decrease of sensitivity (33, 34). However, this has not yet been evaluated for zCIM.

To the best of our knowledge, this is the most comprehensive comparison of commercially available CDTs, and a large number of molecularly characterized CPE isolates were tested. However, CPE carrying rare carbapenemases like IMI, GES, and OXA-58 as well as double-positive CPE were overrepresented (Table S1). This might explain the overall weak performance of the CDT compared to previous studies, most of which included only the most common carbapenemase types. With the inclusion of only KPC, NDM, VIM, IMP, and OXA-48-like CPE, the screening sensitivities would change to 90% (CI, 82 to 95%) for MAST-CDT, 84% (CI, 74 to 90%) for ROS-CDT, and 97% (CI, 91 to 99%) for LIO-CDT. The Ambler class-specific sensitivities would change to 88% (CI, 79 to 94%) for MAST-CDT, 81% (CI, 72 to 89%) for ROS-CDT, and 97% (CI, 91 to 99%) for LIO-CDT.

In conclusion, this study demonstrated the good performance of three commercially available CDTs for the detection and classification of CPE. The tests show strong differences in performance depending on the carbapenemase class. In the diagnostic laboratory, CDT can play a role in screening for CPE in regions with a very limited range of carbapenemases or as an inexpensive test in the case of an outbreak with a carbapenemase that is well detected by this CDT. However, in regions where different carbapenemases prevail, more sensitive and specific tests (e.g., zCIM, colorimetric, immunochromatographic, or molecular assays) should be preferred.
