# Supplementary material for: Systematic Comparison of Three Commercially Available Combination Disc Tests and the Zinc-Supplemented Carbapenem Inactivation Method (zCIM) for Carbapenemase Detection in Enterobacterales Isolates
Source: J Clin Microbiol. 2021 Aug 18;59(9):e03140-20. doi: 10.1128/JCM.03140-20 (PMC8373033; doi:10.1128/JCM.03140-20)
Supplement: Supplemental file 1 — Tables S1 to S3 and Fig. S1. Download JCM.03140-20-s0001.pdf, PDF file, 0.2 MB [file jcm.03140-20-s0001.pdf]

Systematic comparison of three commercially available combination disc tests and zCIM for carbapenemase detection in Enterobacterales isolates

Janko Sattler[a,b], Anne Brunke[a,b], Axel Hamprecht[a,b,c, #]

[a]Institute for Medical Microbiology, Immunology and Hygiene, University Hospital of Cologne, Cologne, Germany

[b]German Centre for Infection Research (DZIF), partner site Bonn-Cologne, Cologne, Germany

[c]Institute for Medical Microbiology and Virology, University of Oldenburg, Oldenburg, Germany

[#]Address correspondence to Axel Hamprecht, Institute for Medical Microbiology, Immunology and Hygiene, University Hospital of Cologne, Cologne, Germany, e-mail: axel.hamprecht@uk-koeln.de, phone: +49 221 478 32009

Running title: Carbapenemase detection by combination disc tests and zCIM

Supplemental material

TABLE S1 Bacterial isolates and carbapenemase classes included in this study. Carbapenemase subtypes comprised GES-25; IMI (-1, -2, -3, -4, -9, -10, -12, -14, -16); KPC (-2, -3); IMP (-1, -4, -8, -22); NDM (-1, -3, -4, -5, -7, -8, -9); VIM (-1, -2, -4, -26, -31, -39, -46, -54, -56, -58); OXA (-48, -162, -181, -204, -232, -244, -245, -370, -58).

	Klebsiella pneumoniae	***E. coli***	***Enterobacter cloacae complex***	***Citrobacter freundii complex***	***Klebsiella aerogenes***	***Serratia marcescens***	***Proteus mirabilis***	***Klebsiella oxytoca***	***Raoultella ornithinolytica***
**Total**	**54**	**44**	**29**	**13**	**6**	**2**	**3**	**1**	**1**
**Carbapenemase positive**	**43**	**25**	**20**	**12**	**0**	**2**	**2**	**1**	**1**
**Class A**	**13**	**0**	**11**	**5**	**0**	**0**	**0**	**0**	**0**
GES	0	0	0	1	0	0	0	0	0
IMI	0	0	9	0	0	0	0	0	0
KPC	13	0	2	4	0	0	0	0	0
**Class B**	**17**	**12**	**9**	**6**	**0**	**2**	**0**	**1**	**1**
IMP	3	0	0	1	0	0	0	0	0
NDM	11	9	4	1	0	1	0	0	1
VIM	3	3	5	4	0	1	0	1	0
**Class D**	**10**	**12**	**0**	**1**	**0**	**0**	**2**	**0**	**0**
OXA-48	2	4	0	0	0	0	0	0	0
OXA-48-like	9	8	0	1	0	0	0	0	0
OXA-58	0	0	0	0	0	0	2	0	0
**Two carbapenemases**	**3**	**1**	**0**	**0**	**0**	**0**	**0**	**0**	**0**
NDM + OXA-48-like	0	1	0	0	0	0	0	0	0
KPC + VIM	2	0	0	0	0	0	0	0	0
**Controls**	**11**	**19**	**9**	**1**	**6**	**0**	**1**	**0**	**0**

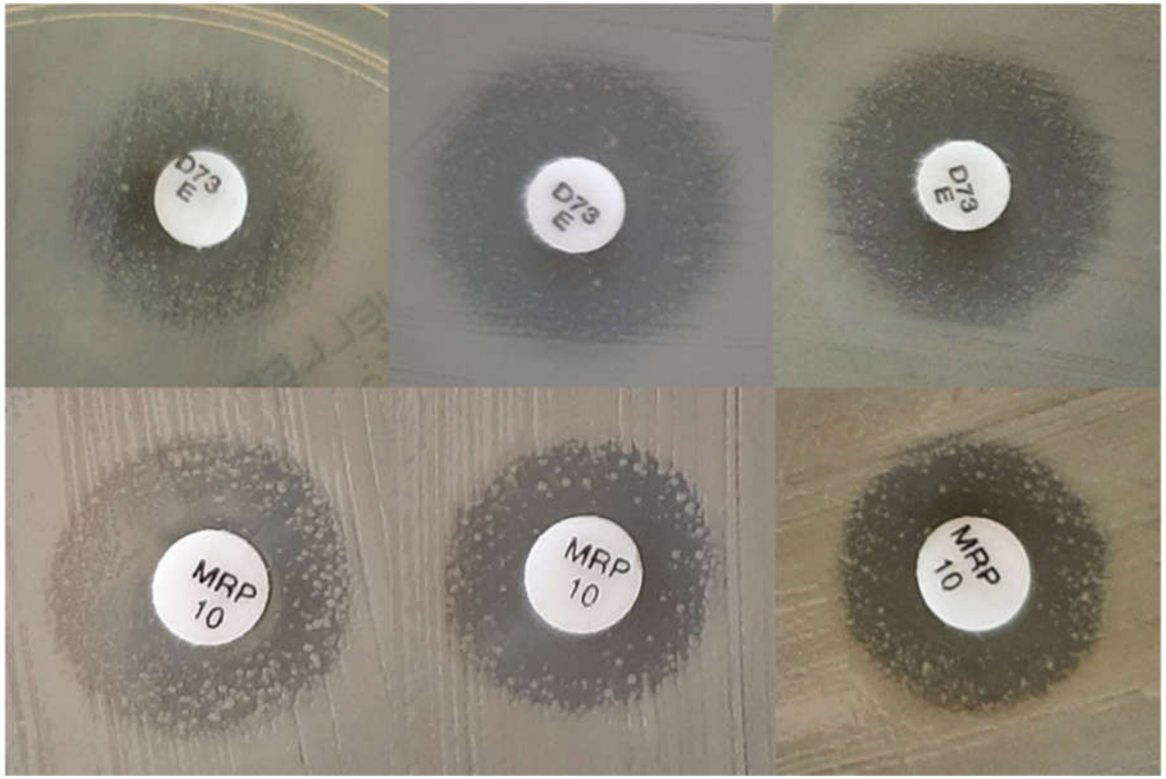

FIG S1 Illustrative examples of microcolonies in the inhibition zone of the temocillin disc of the Mastdiscs Combi Carba plus (top line) and the meropenem disc of the Liofilchem KPC&MBL&OXA-48 disc kit (bottom line) on different Mueller-Hinton agars. From left to right: Oxoid, Axonlab, Becton Dickinson.

TABLE S2 Cut-off values for the examined combination disc tests. The carbapenem + inhibitor values are relative increases of the inhibition zone compared to the carbapenem alone. Temocillin values are absolute values. Periods represent values not taken into account for the respective interpretation. MAST-CDT = Mastdiscs Combi Carba plus, ROS-CDT = Rosco KPC/MBL and OXA-48 Confirm Kit, LIO-CDT = Liofilchem KPC& MBL& OXA-48 disc kit, PBA = phenylboronic acid, DPA = dipicolinic acid.

**MAST-CDT**		Penem + KPC inhibitor	Penem + MBL inhibitor	Penem + AmpC inhibitor	Temocillin + MBL inhibitor
Class A	Penem	≥ 5 mm	< 5 mm	< 5 mm	.
Class B	Penem	< 5 mm	≥ 5 mm	< 5 mm	.
Class D	Penem	< 5 mm	< 5 mm	< 5 mm	≤ 10 mm
**ROS-CDT**		Meropenem + PBA	Meropenem + DPA	Meropenem + Cloxacillin	Temocillin
Class A	Meropenem	≥ 4 mm	≤ 3 mm	≤ 3 mm	.
	Meropenem + Cloxacillin	≥ 4 mm	.	.	.
Class B	Meropenem	≤ 3 mm	≥ 5 mm	≤ 3 mm	.
Class D	Meropenem	≤ 3 mm	≤ 3 mm	≤ 3 mm	≤ 12 mm
**LIO-CDT**		Meropenem + PBA	Meropenem + EDTA	Meropenem + Cloxacillin	Temocillin
Class A	Meropenem	≥ 4 mm	< 5 mm	< 5 mm	.
Class B	Meropenem	< 4 mm	≥ 5 mm	< 5 mm	.
Class D	Meropenem	< 4 mm	< 5 mm	< 5 mm	< 11 mm

TABLE S3 Results of false positive/negative CDT with OXOID MHA when assessed on different Mueller-Hinton agars. Numerators represent correctly identified samples on the respective agar/CDT combination, denominators represent the number of samples that initially yielded a false positive/negative result on OXOID MHA. BD = Becton Dickinson, MAST-CDT = Mastdiscs Combi Carba plus, ROS-CDT = Rosco KPC/MBL and OXA-48 Confirm Kit, LIO-CDT = Liofilchem KPC& MBL& OXA-48 disk kit, FAR = faropenem

	**MAST-CDT**		**ROS-CDT**		**LIO-CDT**		**FAR**	
	Axonlab	BD	Axonlab	BD	Axonlab	BD	Axonlab	BD
**true positive**	1/15	2/15	1/17	1/17	2/4	1/4	0/1	0/1
**true negative**	1/1	1/1	0/1	0/1	1/6	0/6	1/9	0/9
